# Assessment of Developmental and Reproductive Fitness of Dengue-Resistant Transgenic *Aedes aegypti* and Improvement of Fitness Using Antibiotics

**DOI:** 10.1155/2021/6649038

**Published:** 2021-03-02

**Authors:** Hewawasam Patuwatha Badathuruge Kalindu Dulanja Ramyasoma, Yasanthi Illika Nilmini Silva Gunawardene, Menaka Hapugoda, Ranil Samantha Dassanayake

**Affiliations:** ^1^Department of Chemistry, Faculty of Science, University of Colombo, Colombo, Sri Lanka; ^2^Molecular Medicine Unit, Faculty of Medicine, University of Kelaniya, Kelaniya, Sri Lanka

## Abstract

**Background:**

Genetic modification offers opportunities to introduce artificially created molecular defence mechanisms to vector mosquitoes to counter diseases causing pathogens such as the dengue virus, malaria parasite, and Zika virus. RNA interference is such a molecular defence mechanism that could be used for this purpose to block the transmission of pathogens among human and animal populations. In our previous study, we engineered a dengue-resistant transgenic *Ae. aegypti* using RNAi to turn off the expression of dengue virus serotype genomes to reduce virus transmission, requiring assessment of the fitness of this mosquito with respect to its wild counterpart in the laboratory and semifield conditions.

**Method:**

Developmental and reproductive fitness parameters of TM and WM have assessed under the Arthropod Containment Level 2 conditions, and the antibiotic treatment assays were conducted using co-trimoxazole, amoxicillin, and doxycycline to assess the developmental and reproductive fitness parameters.

**Results:**

A significant reduction of developmental and reproductive fitness parameters was observed in transgenic mosquito compared to wild mosquitoes. However, it was seen in laboratory-scale studies that the fitness of this mosquito has improved significantly in the presence of antibiotics such as co-trimoxazole, amoxicillin, and doxycycline in their feed.

**Conclusion:**

Our data indicate that the transgenic mosquito produced had a reduction of the fitness parameters and it may lead to a subsequent reduction of transgenic vector density over the generations in field applications. However, antibiotics of co-trimoxazole, amoxicillin, and doxycycline have shown the improvement of fitness parameters indicating the usefulness in field release of transgenic mosquitoes.

## 1. Introduction

Dengue virus (DENV) is one of such arboviruses carried by Aedine mosquito vectors, which are some of the most dangerous arbovirus carriers worldwide, and *Aedes aegypti* is one of such vectors which transmit dengue, Zika, yellow fever, chikungunya viruses, etc. and is responsible for dengue (DEN) epidemics in more than 100 tropical and subtropical countries [1]. Also, *Ae. aegypti* is adapting to new environments of the other parts of the world and increases the risk of transmission of dengue on those areas [2, 3]. Since there are no vaccines or medicines available for the DENV, DEN control entirely depends on the vector control programs which have so far achieved limited success due to the reduced vector surveillance and control, dissemination of insecticide resistance among vector populations [4], increased urbanization and global travel, and global warming trends that favour the spread of dengue vectors [5]. Therefore, in addition to the conventional vector control strategies, new strategies based on controlling the DENV population have been warranted [6]. Genetic manipulation of mosquito vectors is one of the novel strategies that can be used to control disease-transmitting mosquito vectors. RNA interference- (RNAi-) based approach using synthetically constructed DNA cassettes has been used to block the transmission of DENV and chikungunya virus [7]. The assessment of fitness parameters of transgenic mosquitoes before releasing them into the fields is imperative as it is an important measurement of living modified animals, and it defines the feasibility of using a transgenic approach in a natural environment. Fitness can be divided into developmental and reproductive fitness [8, 9]. The developmental fitness is a key factor that gives an advantage in survival and adapts to the environment, while the reproductive fitness is the ability of individuals to pass on their genes to subsequent generations. Transgenic mosquitoes have lower mating success than wild mosquitoes due to the hitchhiking effect caused by insertional mutagenesis. Wild mosquito poses an evolutionary advantage of fitness than transgenic mosquitoes and this will result in the subsequent reduction of transgenic mosquito proportions in the future generation. Therefore, the examination of the reproductive and developmental fitness of mosquitoes is one of the assessments to be performed before releasing mosquitoes into the fields [10].

In this study, the fitness assessments were carried out for the multiple dengue serotype-resistant *Ae. aegypti* developed by us [11]. The developmental and reproductive fitness assessments of this mosquito were significantly lower than those of wild mosquitoes. Since the fitness is an important factor in the field release, the possibilities to increase the fitness of transgenics were examined in the presence of antibiotics such as co-trimoxazole, amoxicillin, and doxycycline and this showed that these antibiotics can improve the fitness of mosquitoes in laboratory studies indicating adaptability of this in the field release of transgenic mosquitoes.

## 2. Materials and Methods

### 2.1. Mosquito Raring and Maintenance

*Ae. aegypti* wild mosquitoes (WM) and transgenic mosquitoes (TM) were maintained as mentioned in Ramyasoma et al. [11]. Hemizygous TM and subsequent generations of WM were maintained at the Arthropod Containment Level 2 (ACL2) Facility, Molecular Medicine Unit, Ragama.

### 2.2. Measurement of Developmental Fitness Components

The fitness of mosquitoes sampled from cage populations of the hemizygous transgenic strain and the wild *Ae*. *aegypti* Sri Lankan strain was measured. The developmental fitness traits included mosquito oviposition, fertility, body size (measured from head to abdomen apical margin) with their wing lengths, larval life span, adult life span, and temperature sensitivity of larvae. All of these fitness traits were measured in three replicate experiments. The populations and subsequent generations used were reared in the constant conditions as previously described because the traits measured in this study could be sensitive to environmental conditions. The differences observed among the populations and their different filial generations are therefore likely to be due to their genetic differences.

#### 2.2.1. Oviposition

Females were fed with cattle blood as mentioned in Ramyasoma et al. [11], and 100 gravid females (females with 4 days after a blood meal) in three replicates were then transferred to twenty-four cell culture plates for oviposition. Female mosquitoes are allowed to lay eggs for 5 days, and oviposition was scored as the number of eggs laid and any eggs preserved during a single gonotrophic cycle. In some cases, no eggs retained by female mosquitoes were excluded from counting.

#### 2.2.2. Fertility/Hatchability

Collected eggs of the oviposition test were dipped in 50 mL deoxygenated water with a small drop of larval food containing cups, and the eggs were kept for 5 days to emerge larvae. Emerged larvae were counted, and fertility/hatchability was calculated with respect to the collected eggs in the oviposition test.

#### 2.2.3. Larval Life Span

To determine the larval life span, 100 of the first-instar larvae of the founder populations and their filial generations were transferred into distilled water maintained at a white tray having the size of 15′5^″^ × 11′5^″^ × 2′5^″^. Trays containing larvae were fed daily with IAEA-recommended larval food diet [12] and they were subjected to artificial light and were monitored daily. Pupated larvae were recorded and then were transferred to emergence cages, and the number of dead larvae was also recorded and removed.

#### 2.2.4. Adult Life Span and Survival Curves

Adult life span was synchronized by selecting a batch that emerged on the same day (day 0), and adult life span and survival rates were calculated by counting dead mosquitoes with days. A portion of 200 male and female pupae of TM and WM were placed separately in cages in the insectary and they were maintained as previously described. Mosquitoes were examined daily, and the dead individuals were counted and removed until the day that the last individual is died.

#### 2.2.5. Body Length

The body length of female TM and WM individuals (*n* = 100) was measured. Body length was measured from head to abdomen apical margin and was measured to the nearest 0.05 mm using a microscope ruler reticle scale micrometer (ZZCAT, China) and a compound microscope.

#### 2.2.6. Wing Length

The wing lengths of the females and males were measured from TM and WM individuals (*n* = 100) by mounting them on a glass microscope slide in a small drop of distilled water. Wing length was measured to the nearest 0.05 mm using a microscope ruler reticle scale micrometer (ZZCAT, China) and a compound microscope.

#### 2.2.7. Larva Activity under Different Water Temperatures

Thirty larvae of *Ae*. *aegypti* placed at each larval tray set at 15°C, 20°C, 25°C, 30°C, 35°C, 40°C, and 45°C by the mechanical thermometer were analyzed for their activity. The fourth-instar larvae of mosquitoes were used in the experiments keeping them in a constant light intensity. Larvae immobilized in a cold environment were reactivated by transferring them into larval trays maintained at the abovementioned temperatures. After placing larvae in the first 10 min, actively swimming larvae were counted using a pipette and care was taken to not to count actively swimming larvae more than one time. This procedure was repeated for 30 times under the same conditions of light and humidity, and both TM and WM larvae were used in the experiment. The room temperature at 28°C was taken as the control of the experiment.

#### 2.2.8. Mating Competitiveness of Adult Mosquitoes in the Insectary and Semifield Trials

The experiments were carried out under laboratory and semifield conditions. Insectary conditions were adjusted to maintain the room temperature and relative humidity of 28°C and 70%-80%, respectively. Environmental conditions of 25-31°C room temperature and 65-84% relative humidity were used as semifield conditions for semifield experiments. Mating combinations of C (WM♂ : WM♀ = 1 : 1), G1 (TM♂ : WM♀ = 1 : 1), G2 (WM♂ : TM♀ = 1 : 1), G3 (WM♂ : TM♂ : TM♀ = 1 : 1 : 1), and G4 (TM♂ : WM♂ : WM♀ = 1 : 1 : 1) were placed in laboratory adult cages or large semifield cages and kept overnight to facilitate mating, and mosquito eggs were collected after feeding them with cattle blood for 45 min. Each mating combination had 100 males and females of TM and WM except for the control which had only wild (WM ♂ : WM ♀) combination, and these experiments were triplicated. A filter paper with water in a cup was placed to facilitate females to lay their eggs, the collected eggs from each combination were allowed to hatch in distilled water with larval food, and the number of L1 larvae obtained was recorded. TM rates were calculated by observing emerged L4 larvae of laboratory (randomly selected 10% larvae to the total) and emerged L4 larvae of semifield experiments under the Olympus BX53 fluorescent microscope, and the relative mating success is calculated as the (resulted TM rate)/(100-resulted TM rate) from resulted progeny.

### 2.3. Antibiotic Effects on Larval Life Span and Adult Life Span

Adults mosquitoes were fed with syrups, and larvae were exposed to water containing antibiotics such as amoxicillin (Axil 250, Astron Ltd., Sri Lanka), doxycycline (Leben Laboratories Pvt. Ltd., India), and co-trimoxazole (Fourrts Laboratories Pvt Ltd., India) while maintaining them at a room temperature and 70-80% relative humidity. The minimum inhibitory concentrations of amoxicillin (0.25 *μ*g/mL) [13], doxycycline (5 *μ*g/mL), and co-trimoxazole (2.5 *μ*g/mL for trimethoprim and 60 *μ*g/mL for sulfamethoxazole) [14] were used for larval water, adult syrup, and cattle blood to analyze oviposition, fertility, larval life span, and adult life span as mentioned previously. Larval water, adult syrup, and cattle blood without antibiotics were used as the control of the experiment.

### 2.4. Antibiotic Effect on Adult Mating between TM and WM

Differential mating was carried out by placing 100 male and female adult mosquitoes in the following combinations: AC (WM♂ : WM♀ = 1 : 1), AG1 (WM♂ : TM♀ = 1 : 1), and AG2 (TM♂ : WM♂ : WM♀ = 1 : 1 : 1) while allowing them to mate. The total egg count and fertility were measured, and TM proportions were counted by observing randomly selected 10% emerged L4 larvae under the Olympus BX53 fluorescent microscope.

### 2.5. Data Analyses

All the experiments described here were triplicated. Data obtained for oviposition, fertility, larval life span, adult body length, and adult wing length parameters were compared using the Mann–Whitney *U* test, and adult survival curves were analyzed using the log-rank (Mantel-Cox) test. The D'Agostino and Pearson normality test was used to analyze the distribution of the adult body and adult wing length data. The Kruskal-Wallis test was followed by Dunn's multiple comparison test was used to assess active larva count of different temperatures. Antibiotic treatment data obtained for oviposition, fertility, and larval life span parameters were compared using the Kruskal-Wallis test and followed by Dunn's multiple comparison test, and adult survival curves were analyzed using the log-rank (Mantel-Cox) test. The chi-square test was used to assess the fertility rate and TM rate of the mating competitiveness experiment and antibiotic-treated experiment. Data analysis was conducted using the GraphPad Prism 7 Software Package (Version 7.04) for Windows (San Diego, California, USA), and confidence intervals of 95% were defined for all analyses.

## 3. Results

### 3.1. Developmental Fitness Component Assessment

**Figure 1:**
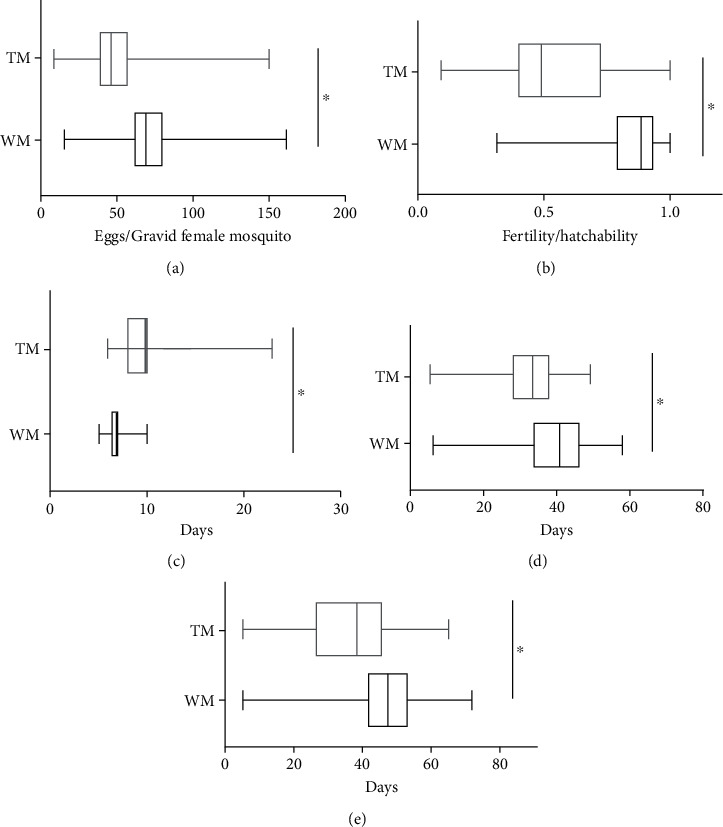
Developmental fitness parameter measurements of transgenic mosquito (TM) in comparison to wild mosquitoes (WM): (a) oviposition, (b) fertility, (c) larval life span, (d) adult male life span, and (e) adult female life span. Survival curves were compared between WM and TM. Approximately, 200 mosquitoes of TM and WM were used in this experiment. Oviposition defined as the number of eggs per blood-fed female mosquitoes and female mosquitoes without any eggs laid was excluded from results; fecundity as the number of L1 larvae/number of eggs; larval life span as the number of days to become pupae from larvae; and adult life span as the number of days to the death of the mosquitoes. Three replicates were performed for each experiment. The Mann–Whitney *U* test was performed to analyze the parameters between WM and TM. Bars represent maximum value to the minimum value of the results of TM or WM. Significant differences at *p* < 0.05 are indicated by ∗.

**Figure 2:**
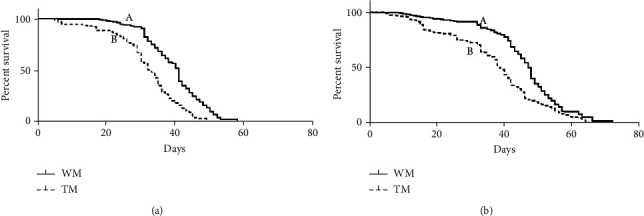
Survival curves of WM and TM: (a) males and (b) females. Survival curves were compared between WM and TM. Approximately, 200 mosquitoes of TM and WM were used in this experiment (TM: transgenic mosquito; WM: wild mosquito). Survival curve statistical analysis was performed using a log-rank (Mantel-Cox) test.

To determine whether the transgene has any significant effect on the fitness, the hemizygous TM line, SL1161, was compared with the WM *Ae. aegypti* colony in a laboratory trial using fitness parameters, such as oviposition, fertility, larval life span, and adult male and female life span (Figure 1). Female oviposition for TM was 50.98 ± 1.80 (*n* = 168), and this was significantly lower than that of WM, 77.62 ± 1.988 (*n* = 188) (Mann–Whitney *U* test: *p* < 10^−4^). Similarly, the average hatchability of eggs/fertility for TM was 0.55 ± 0.02 (*n* = 168) and this was lower than that of WM at 0.84 ± 0.01 (*n* = 188) (Mann–Whitney *U* test: *p* < 10^−4^). The average life span of adult male TM was 31.83 ± 8.94 days and this was significantly shorter than that of WM, 39.71 ± 10.04 days. The average life span of female TM was 37.02 ± 13.46 days and this was shorter than that of WM, 45.54 ± 12.70 days. Also, survival curves of adult male and female TM were significantly different from those of WM (Figure 2, log-rank (Mantel-Cox) test: *p* < 10^−4^).

### 3.2. Mosquito Body Length and Wing Length

Mosquito body and wing lengths are vital measurements in developmental fitness. The average body length of male mosquitoes did not show a significant difference between the WM and TM (Table 1, Mann–Whitney *U* test, *p* < 0.05) and similarly, the average body length of TM female mosquitoes and WM females (Mann–Whitney *U* test, *p* < 0.05). Further, the average wing length measurements of TM females did not show a significant difference to WM females (Mann–Whitney *U* test, *p* < 0.05), as well as average wing length measurements of male TM from WM (Table 1, Mann–Whitney *U* test, *p* < 0.05).

### 3.3. Larva Activity under Different Water Temperatures

**Figure 3:**
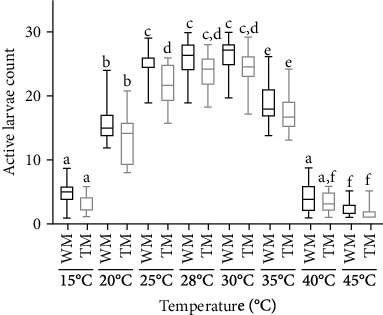
Activeness measurements of L4 larvae of TM in comparison to WM under different temperatures. The Kruskal-Wallis test was performed to analyze the parameters between WM and TM. Bars represent maximum value to the minimum value of the results of TM or WM. Data points followed by the same lowercase letter do not differ significantly from each other by Dunn's test (*p* < 0.05).

Fourth-instar larvae of both TM and WM showed a marked preference for the temperatures of 25°C, 28°C, and 30°C while showing lower active larva rates in temperatures of 15°C, 20°C, 35°C, 40°C, and 45°C (Figure 3). The measurement average active larva rate of WM at temperatures of 20°C and 25°C is 40.89 ± 1.74 and 72.78 ± 1.87, respectively, and was significantly higher than TM and was 52.11 ± 1.78 and 83.67 ± 1.44 for 20°C and 25°C, respectively (Dunn's, *p* < 0.05). The highest average active larva count temperature was observed at 30°C, and the lowest average active larva count was observed at 45°C.

### 3.4. Mating Competitiveness between TM and WM in Insectary Conditions and Semifield Conditions

The outcomes of mating competitiveness experiments in both laboratory and semifield cages are presented as fertility and TM percentage (Table 2). The mating mixes of C, G1, and G3 in both laboratory and semifield experiments showed a significantly higher fertility rate than other mating mixes of G2 and G4 (Table 2, chi-square, *p* < 0.05 (laboratory and field assays)), as well as results showed that the TM rates for mating mixes of G1 and G2 were not significantly different from each other under laboratory and semifield conditions. Field release of the G3 mating mix showed a reduced TM proportion than other mixes in semifield and laboratory assays (chi-square test, *p* < 0.05 (laboratory and field assays)).

### 3.5. Antibiotic Effects on Developmental Fitness Measures of TM and WM

**Figure 4:**
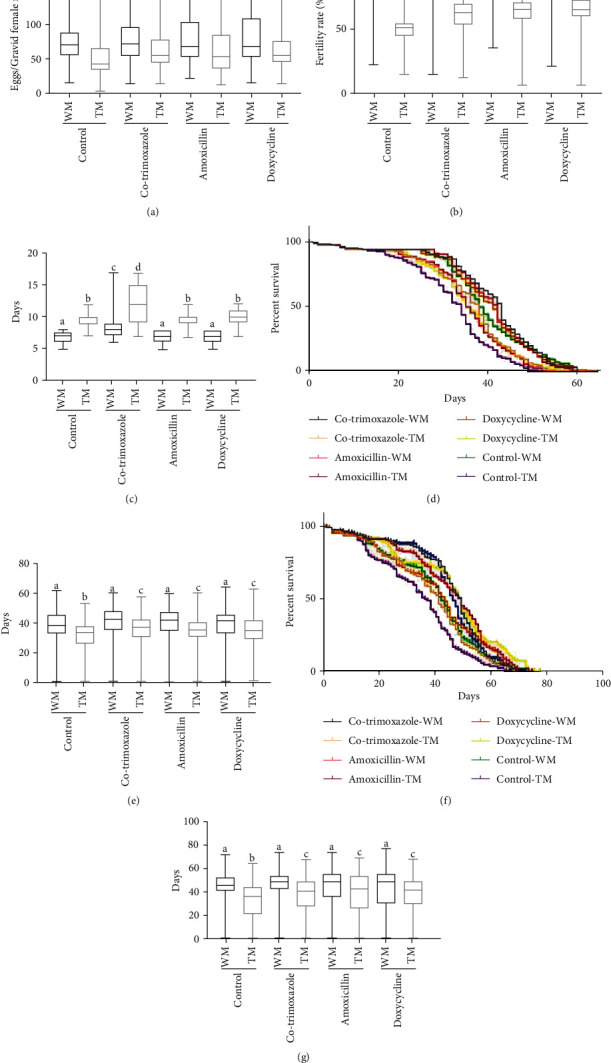
Developmental fitness measures of antibiotic-treated wild mosquitoes (WM) and transgenic mosquitoes (TM): (a) oviposition, (b) fertility, (c) larval life span, (d) adult male survival curves, (e) adult male life span, (f) adult female survival curves, and (g) adult male life span. Oviposition defined as the number of eggs per blood-fed female mosquitoes and female mosquitoes without any eggs laid was excluded from results; fecundity as the number of L1 larvae/number of eggs; larval life span as the number of days to become pupae from larvae; and adult life span as the number of days to the death of the mosquitoes. Three replicates were performed for each experiment. Data points followed by the same lowercase letter do not differ significantly from each other by Dunn's test (*p* < 0.05). Survival curve statistical analysis was performed using a log-rank (Mantel-Cox) test. Bars represent maximum value to the minimum value of the results of TM or WM.

Oviposition, fertility, larval life span, and adult life span measurements were assessed for both TM and WM in the presence of antibiotics. Antibiotic treatments of TM samples have shown significant improvement in oviposition measures compared to its nontreated control of TM (53.66 ± 3.09, 63.31 ± 3.04, 63.97 ± 3.87, and 62.46 ± 2.96 for control, co-trimoxazole, amoxicillin, and doxycycline, respectively, Figure 4(a), Dunn's test, *p* < 0.05). However, the improvement of the oviposition of antibiotic-treated TM did not significantly equal to WM (79.97 ± 3.89, 81.08 ± 3.80, 80.59 ± 3.90, and 81.57 ± 3.99 for control, co-trimoxazole, amoxicillin, and doxycycline, respectively, Figure 4(a), Dunn's test, *p* < 0.05). Fertility measures of the antibiotic-treated TM were significantly higher compared its nontreated control of TM (52.74 ± 1.87, 62.08 ± 2.02, 63.3 ± 2.18, and 63.58 ± 2.09 for control, co-trimoxazole, amoxicillin, and doxycycline, respectively, Figure 4(b), Dunn's test, *p* < 0.05), and also the improvement of treated TM was not significantly equal to WM (80.19 ± 1.46, 85.53 ± 1.18, 83.46 ± 1.43, and 83.12 ± 1.31 for control, co-trimoxazole, amoxicillin, and doxycycline, respectively, Figure 4(b), Dunn's test, *p* < 0.05). The larval life span of the co-trimoxazole-treated sample of WM showed a significant increase of life span value than the other antibiotic-treated and antibiotic-nontreated samples of WM (8.871 ± 0.29 and 6.881 ± 0.08 days for co-trimoxazole-treated and co-trimoxazole-nontreated WM, Dunn's test, *p* < 0.05). Further, the significant effect on larval life span values from amoxicillin and doxycycline (Dunn's test, *p* < 0.05) was not seen. Adult male life span measures of TM treated with antibiotics were 35.31 ± 0.80, 34.65 ± 0.77, and 34.63 ± 0.80 days for co-trimoxazole, amoxicillin, and doxycycline, respectively, and showed a significant improvement to nontreated TM (31.58 ± 0.75 days), but the improvement was not significantly equal to WM (Dunn's test, *p* < 0.05). Also, the same was observed in adult female life span measures. Antibiotic-treated TM female life spans were 38.09 ± 1.10, 39.95 ± 1.24, and 39.27 ± 1.13 days for co-trimoxazole, amoxicillin, and doxycycline, respectively, and showed the measures were significantly increased than nontreated TM (33.79 ± 1.08 days) (Dunn's test, *p* < 0.05), but the improvements were not significantly equal to WM (Dunn's test, *p* < 0.05).

### 3.6. Antibiotic Effect on Adult Mating between TM and WM

Antibiotic treatments of mating mixes of AG1 and AG2 have shown a significant increase in their fertilities compared to their control samples of nontreated TM and WM (Table 3, chi-square test, *p* < 0.05). Also, the mean TM rates of antibiotic-treated AG2 mix samples (field release scenario) have shown significant improvement compared to AG2 nontreated mix (Table 3, chi-square test, *p* < 0.05 (laboratory and field assays)).

## 4. Discussion

The field release of mosquitoes having disease-resistant phenotypes is one of a specific application and promising option to be used in the future vector-borne disease control [15–17]. These mosquitoes mate with wild populations and produce transgenic offspring with disease-resistant phenotype to reduce disease transmission among the human population [18]. Since the genome of transgenic mosquitoes is manipulated, their fitness is often less compared to natural wild populations and therefore the assessment of TM fitness is key to assess the behaviour and success of the TM applications. Oviposition, fertility, larval life span, adult life span, and mating competitiveness are the major fitness parameters of developmental and reproductive fitness to be assessed in field releasing applications [19, 20]. In addition to the above parameters, in this study, mosquito activities on temperatures, wing length, and mosquito body length were also taken as the developmental fitness parameters to assess the fitness of the TMs. Fitness parameter assessments of TM mosquitoes had shown a significant reduction compared to native wild mosquitoes, which are common to transgenic organisms due to genomic alterations [21].

Oviposition is a vital fitness trait, which reflects the efficiency of the conversion of the blood meal to egg production and then to embryo [22]. Also, it influences the number of offspring that can be produced by a female mosquito [23]. Oviposition results between TM and WM showed significant differences. TM had lower oviposition compared to WM (Figure 1(a), Mann–Whitney *U* test, *p* < 0.05), indicating the conversion of the blood into mosquito eggs not as efficient as in TM compared to WM. Further, a significant difference between TM and WM (Figure 1(b), Mann–Whitney *U* test, *p* < 0.05) in fertility was seen and the reduced fertility and oviposition in TM compared to WM may have led to a subsequent reduction of transgenic phenotype in mosquito populations. The survival of larvae in urban environments influences the larval life span because urban areas' water containers drain within a few days and larvae with least life span are survived [24]. The larval life span of TM showed a significant increase of the life span compared to WM (Figure 1(c), Mann–Whitney *U* test, *p* < 0.05), and therefore, TM may not be competitive and adaptive in urban areas compared to WM as the longer the time mosquito takes to emerge as adults, the lesser are the chances of survival [10]. The adult stage is crucial for mosquitoes because during this period, they mate to produce the next generation of mosquitoes and the adult life span positively affects the mating frequency and increase of gonotrophic cycles of females [25]. Hence, adult mosquitoes with higher life spans acquire the fitness advantage than other mosquitoes. The survival curve and life span measures of adult females and adult males of TM were significantly lower than those of WM (Figures 3(d) and 3(e), *p* < 0.05) and this may also lead to the reduction of transgenic phenotype in mosquito populations. The wing length and female body lengths of the mosquito are the vital parameters that influence survivorship and the ability to acquire a blood meal [17, 24]. TM and WM had no significant difference in fitness parameters of wing lengths and body lengths (Table 1, Mann–Whitney *U* test, *p* < 0.05), and therefore, TM may not be having disadvantages in survivorship and blood feeding caused by wing lengths and body length.

Mosquitoes are cold-blooded animals; they cannot regulate the body temperature and depend on the environmental temperatures, and therefore, the latter acts as an external factor of the fitness of the mosquitoes [26, 27]. The highest activity of larvae was found between the temperature ranges of 25°C-30°C. However, the larval activity measurements of TM at different temperatures showed a significant difference to WM at 20°C and 25°C, but not at 28°C and 30°C, and these variations in low temperature may be disadvantageous for field applications of TM in cold environmental conditions (Figure 3, Mann's test, *p* < 0.05).

Mating competitiveness is a major assessment of reproductive fitness, which demonstrates the ability to transfer their phenotypes to the next generation. Mating competitiveness was assessed with two parameters of fertility, i.e., TM rate and relative mating success. Fertility measurements of the mating mix of WM♂ : TM♂ : TM♀ (G4) and WM♂ : TM♀ (G2) were significantly less when compared with other mating mixes (G1 and G3), demonstrating that TM♀ was not that fertile as WM♀ and this perhaps due to the consumption of amino acids for the production of the endogenous reporter protein in mosquitoes leaving less amount of proteins for embryos and progeny development [19]. The relative mating success of TM was calculated to compare the Mendelian theoretical values of mating success (Table 2). If TM and WM males have an equal mating chance with WM female, they produced 25% of TM progeny to which the Mendelian theoretical relative mating success value of TM is 25/(100 − 25) = 0.33; however, it has been found the mating success value of mating mix, G3, was lower than 0.33 (<0.33) suggesting WM♂ are more competitive in mating than TM♂ in the laboratory and semifield trials. As per the Mendelian theory, if TM mate with WM, they should produce 50% TM progeny for which theoretical relative mating success of TM is 50/(100 − 50) = 1 [28, 29], and however, experimental relative mating success obtained for G2 and G3 was less than 1 (<1.0) and this reflects a situation that there is a reduction of TM over the generations due to reduction of fertilities of TM eggs. In the G4 mating mix, if TM and WM males have an equal chance of mating with TM female, they should produce 75% TM progeny to which theoretical relative mating success of TM should have a value of 75/(100 − 75) = 3. However, the experimental relative mating success values of G4 mating mix were <3, demonstrating the effects of WM male dominance in matings and the reduction of fertility in TM eggs and these assess the developmental fitness measures of mosquitoes.

The fitness measures showed a significant reduction of the latter in TM compared to WM and this may have less success in the field trials, and subsequent release of mosquitoes is required to establish the resistant gene in wild population [19, 30]. One of the reasons for the reduction of fitness may be due to pathogens mosquitoes carry and diseases that they caused and this can be improved by using antibiotics in feeding [14, 19, 20, 30]. Therefore, the antibiotic effects in fitness have been tested with co-trimoxazole, amoxicillin, and doxycycline. These antibiotic treatments were shown to increase oviposition, fertility, and adult life span of TM compared to nontreated TM (Figure 4, Kruskal-Wallis test followed by Dunn's test, *p* < 0.05). However, these increases were not equal to WM indicating antibiotic treatments along will not be able to achieve the fitness of TM to the level of WM. On the other hand, the larval life span of co-trimoxazole antibiotic-treated TM mosquitoes showed a significant increase compared to the other two antibiotic-treated samples (Figure 4(c), Kruskal-Wallis test followed by Dunn's test, *p* < 0.05) suggesting the folate deficiency that caused by co-trimoxazole in folate synthesis pathway prolongs the life span of larvae [31–33]. Further, the effect of antibiotic on mating competitiveness showed a significant increase in the TM progeny percentage of field release scenario mix (Table 3, chi-square test, *p* < 0.05) of the laboratory trials. However, antibiotic-treated TM samples were not able to match the fitness of the nontreated WM indicating that external factors/parameters other than antibiotics that increase the fitness of TM will have to be looked for.

## 5. Conclusions

The fitness cost of TM mosquitoes is an important factor to be considered in-field release and its success. Not like sterile transgenic mosquitoes, the disease-resistant transgenic mosquitoes compete with their wild population to increase their disease-resistant gene frequency in their progeny to reduce the disease transmission among humans. The developmental and reproductive fitnesses are important parameters to be considered in transgenic mosquito-based disease control strategies. The transgenic mosquito had lower developmental and reproductive fitness compared to WM, which is common to transgenic mosquitoes, but this is a disadvantage of field release transgenic mosquitoes. Therefore, the subsequent release of transgenic mosquitoes is required to replace the wild population of mosquitoes unless expected results of the resistant mosquitoes may not be able to achieve and prolonged results cannot be achieved due to the reduction of transgenic proportion over the generations. Further, the study was able to show that antibiotics co-trimoxazole, amoxicillin, and doxycycline can improve the fitness in laboratory-scale studies and it will be useful in the release of TM into the fields.

## Figures and Tables

**Table 1:** Comparison of adult body size and adult wing size of transgenic and wild mosquitoes of *Aedes aegypti.*

	TM		WM	
	Male	Female	Male	Female
Adult body size	3.01 ± 0.01 (*n* = 97)	3.76 ± 0.01 (*n* = 92)	3.02 ± 0.01 (*n* = 98)	3.77 ± 0.01 (*n* = 97)
Adult wing size	2.02 ± 0.01 (*n* = 100)	2.55 ± 0.01 (*n* = 100)	2.03 ± 0.01 (*n* = 100)	2.57 ± 0.01 (*n* = 100)

Data are presented as the mean ± SEM. No significant differences were observed by the Mann–Whitney test between the wild mosquito (WM) and the transgenic mosquito (TM). D'Agostino and Pearson normality test results for adult male size-WM (K2: 16.73, *p* value: 0.0002), TM (K2: 24.14, *p* value < 0.0001); for adult female size-WM (K2: 85.77, *p* value: 0.0001), TM (K2: 15.61, *p* value < 0.0004); for adult male wing size-WM (K2: 15.01,*p* value: 0.0006), TM (K2: 11.63, *p* value < 0.003); and for adult female wing size-WM (K2: 8.117, *p* value: 0.0173), TM (K2: 13.57, *p* value < 0.0011).

**Table 2:** Fertility and mating success of mating mixes of transgenic and wild mosquitoes (TM and WM) of *Ae. aegypti.*

Mating mix	Laboratory conditions			Semifield conditions		
	Fertility (%)	TM rate (%)	Relative mating success	Fertility (%)	TM rate (%)	Relative mating success
C	82.35 (*n* = 16,305)	—		73.37 (*n* = 2,576)	—	
G1	63.66 (*n* = 14,128)	41.89^b^ (*n* = 899)	0.72	44.15 (*n* = 2,084)	41.74^d^ (*n* = 460)	0.72
G2	54.74^a^ (*n* = 6,136)	41.17^b^ (*n* = 336)	0.70	35.56^c^ (*n* = 838)	40.27^d^ (*n* = 149)	0.67
G3	69.17 (*n* = 16,464)	17.40 (*n* = 1,139)	0.21	60.27 (*n* = 2,648)	16.17 (*n* = 798)	0.19
G4	53.56^a^ (*n* = 6,440)	52.07 (*n* = 345)	1.09	37.50^c^ (*n* = 832)	51.92 (*n* = 156)	1.08

Lowercase letters are showing significant equal rates (chi-square test) between the wild mosquito (WM) and the transgenic mosquito (TM) mating mixes. ^∗^Relative mating success is calculated as the ratio of the percentage of progeny from transgenic males to wild-type males; for example, for 46% TM rate, relative mating success is calculated as 46/(100-46). The number of total eggs or larvae for each experiment is given in parenthesis. Mating mixes are C (WM♂ : WM♀ = 1 : 1), G1 (TM♂ : WM♀ = 1 : 1), G2 (WM♂ : TM♀ = 1 : 1), and G3 (WM♂ : TM♂ : TM♀ = 1 : 1 : 1).

**Table 3:** Fertility and mating success of mating mixes with the treatment of antibiotics of (TM and WM) *Ae. aegypti.*

	Fertility rate (%)			TM rate (%)			Relative mating success		
	AC	AG1	AG2	AC	AG1	AG2	AC	AG1	AG2
Control	83.89 (*n* = 18,735)	63.43 (*n* = 11,400)	69.81 (*n* = 11,404)	—	40.64 (*n* = 723)	17.79 (*n* = 796)	—	0.68	0.22
Co-trimoxazole	83.48 (*n* = 19,823)	65.69^∗^ (*n* = 16,360)	74.77^∗^ (*n* = 14,323)	—	42.75^∗^ (*n* = 1,075)	20.93^∗^ (*n* = 1,071)	—	0.75	0.26
Amoxicillin	83.82 (*n* = 19,295)	65.34^∗^ (*n* = 15,800)	75.08^∗^ (*n* = 14,729)	—	42.69^∗^ (*n* = 1,032)	20.29^∗^ (*n* = 11,058)	—	0.74	0.25
Doxycycline	85.38^∗^ (*n* = 19,375)	66.42^∗^ (*n* = 16,160)	73.95^∗^ (*n* = 14,424)	—	43.41^∗^ (*n* = 1,073)	19.88^∗^ (*n* = 1,067)	—	0.77	0.25

The number of total eggs or larvae for each experiment is given in parenthesis. ∗ is showing significant different to control rates (*p* < 0.05 between transgenic and wild mosquitoes (TM and WM)). Mating mixes are AC (WM♂ : WM♀ = 1 : 1), AG1 (WM♂ : TM♀ = 1 : 1), and AG2 (TM♂ : WM♂ : WM♀ = 1 : 1 : 1).

## Data Availability

The data used to support the findings of this study are available from the authors upon request.

## Abbreviations

TM: Transgenic mosquito
WM: Wild mosquito
RNAi: RNA interference
DENV: Dengue virus
DEN: Dengue
IAEA: International Atomic Energy Agency
